# Renal Involvement in IPEX Syndrome With a Novel Mutation of *FOXP3*: A Case Report

**DOI:** 10.3389/fgene.2021.752775

**Published:** 2022-02-02

**Authors:** Ruijuan Ke, Ying Zhu, Fang Deng, Daliang Xu

**Affiliations:** Anhui Provincial Children’s Hospital, Hefei, China

**Keywords:** IPEX syndrome, FOXP3, diagnosis, treatment, whole-exon sequencing

## Abstract

The immune dysregulation, polyendocrinopathy, enteropathy, X-linked (IPEX) syndrome is a rare genetic disease characterized by multiple immune disorders. Different mutations of the *FOXP3* gene may lead to distinct clinical manifestations. Here, we present a rare case of IPEX syndrome caused by a novel variant of *FOXP3*. Clinical manifestations include autoimmune hemolysis, bronchiectasis, diarrhea, and proteinuria but without diabetes or other endocrine disorders. The diagnosis of IPEX syndrome was confirmed by whole-exon sequencing. Supportive treatment did not ameliorate the patient’s symptoms, while immunosuppressive therapy showed a promising efficacy. The patient we reported will improve the understanding of renal manifestations in IPEX syndrome.

## Introduction

The immunodysregulation, polyendocrinopathy, and enteropathy, X-linked (IPEX; OMIM:#304790) is a rare genetic immunologic disorder with various clinical symptoms, which is caused by mutations in the *FOXP3* gene (Gambineri et al., 2018). The onset of IPEX varies from the natal period to adulthood. Features may include refractory diarrhea, endocrine disorder, and skin disease, which are induced by the dysregulation of CD4^+^CD25^+^ regulatory T (Treg) cells (Bennett et al., 2001; Barzaghi et al., 2012). Treg cells can maintain immune tolerance to self-antigens via inhibiting activities of effective T cells. Therefore, dysregulation will lead to a multitude of clinical symptoms. Due to its X-linked recessive manner, IPEX is inherited in males, leading the boy typically die within the first 2 years if untreated (Barzaghi et al., 2018). Currently, bone marrow transplantation is the only therapeutic approach for IPEX (Ben-Skowronek, 2021).

Due to the clinical heterogeneity and low incidence of IPEX, missed and inaccurate diagnoses are common, resulting in high mortality. Herein, we report a boy who presents typical renal manifestations and carries a novel mutation in *FOXP3* with IPEX syndrome.

## Case Presentation

**FIGURE 1:**
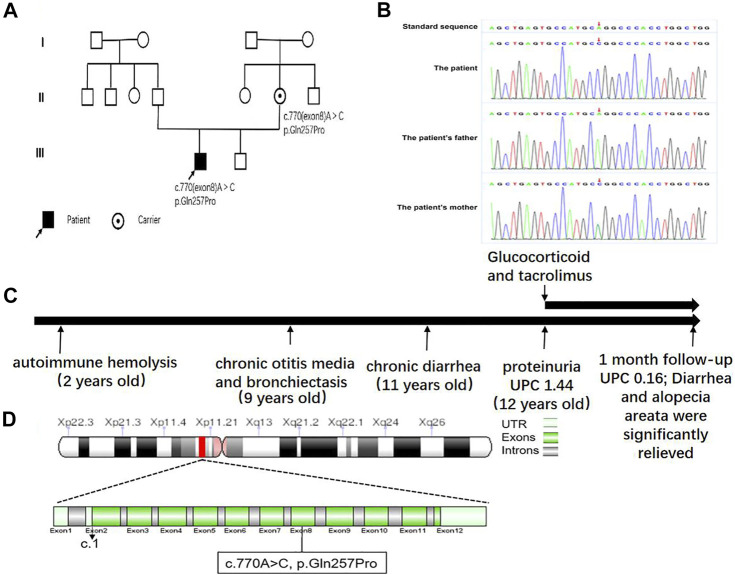
**(A)** Family pedigree of the patient. **(B)** Electrophoretogram of the detected novel pathogenic variant in comparison with the wild-type FOXP3 sequence. **(C)** A schematic diagram of the disease course. UPC, urine protein–creatinine. **(D)** FOXP3 mutation analysis.

**FIGURE 2:**
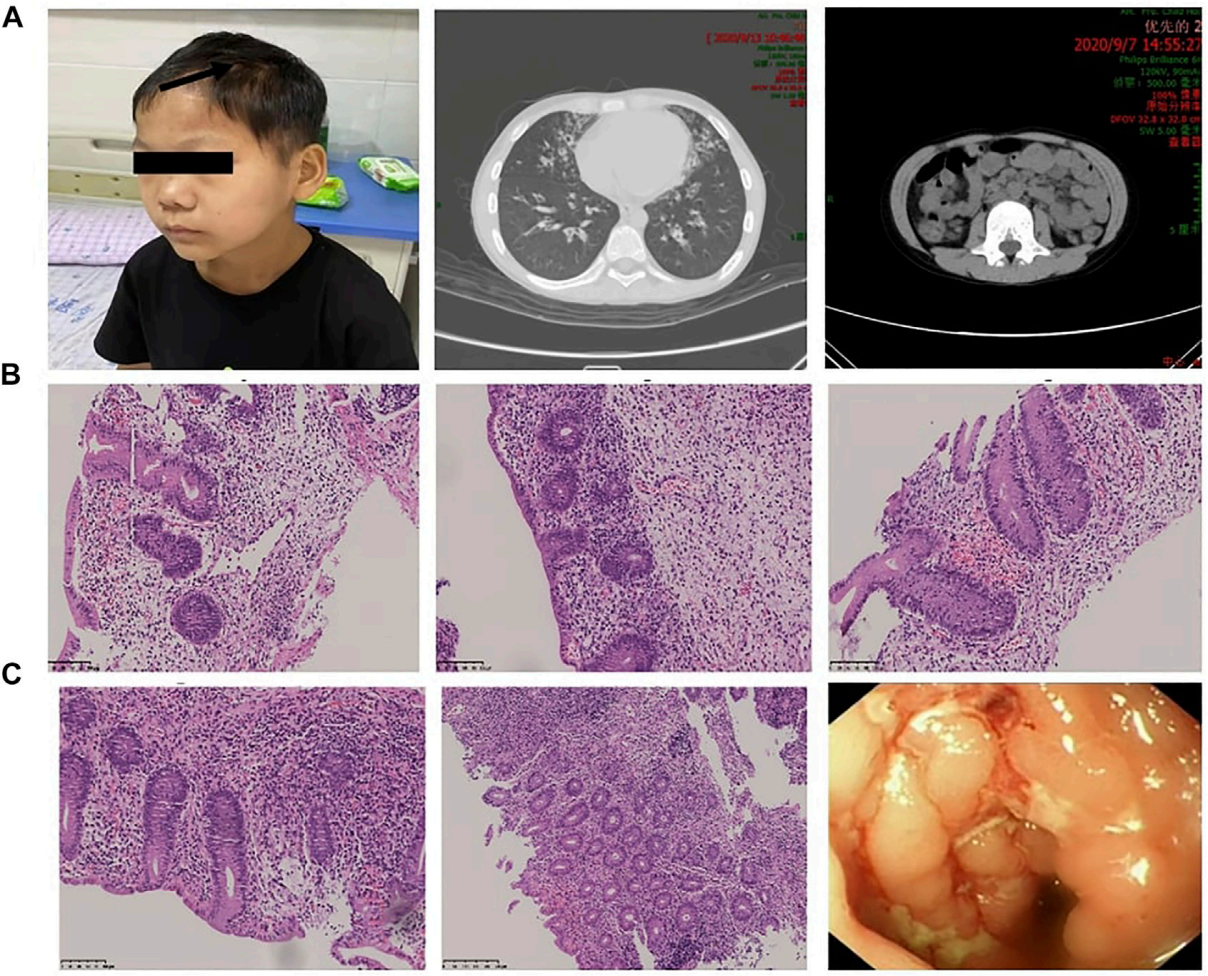
Manifestations caused by autoimmune disorders in different organ systems. **(A)** The patient presented with alopecia areata. Chest CT showed signs of bronchiectasis, and abdominal CT showed swollen and thickened ileocecal and colon walls and enlarged lymph nodes. **(B,C)** Colonoscopic findings and pathological changes in different parts of the intestine (ileocecal junction, ascending colon, descending colon, sigmoid colon, rectum). Magnification: **(B,C)** ×200–400.

A 12-year-old boy was admitted to our department due to proteinuria. He had a remarkable past medical history (Figure 1C): he was diagnosed with autoimmune hemolysis 10 years ago, chronic otitis media and bronchiectasis 3 years ago, and repeated diarrhea and alternative constipation with hair loss in recent years (Figure 2A). 2 weeks ago, his diarrhea aggravated to 5–6 times per day, with medium volume of dilute watery stools, with no blood and stench. The application of oral probiotics and montmorillonite powder failed to ameliorate his symptoms. Therefore, he was admitted to our hospital and transferred to our department because of a strong positive (“++++”) urine protein. His family members including his younger brother have no similar symptoms. We inquired the family history of the boy in detail and found no relatives within three generations had similar clinical manifestations. The pedigree is shown in Figure 1A.

Physical examination revealed that body height (141.0 cm) and weight (32.4 kg) were smaller than those of contemporary children. He had an anemic appearance with alopecia areata, purulence in the bilateral external auditory canal, hearing loss, rales and wheezing sounds in both lungs, and hyperactive bowel sounds. Lab examinations indicated that the white blood cell and CD3^−^CD19^+^ B cells were elevated, whereas the red blood cell (RBC), hemoglobin, and CD3^−^CD16^+^/CD56^+^ NK cells were decreased (Table 1). Autoimmune antibodies had no positive results. Morphology examination reported several oval heteromorphic RBCs with a positive Coombs test. Erythropoietin (EPO, 42.6 IU/L) was increased, and serum transferrin was decreased (1.55 g/L). Meanwhile, the erythrocyte sedimentation rate (ESR, 68 mm/h), procalcitonin (PCT, 0.049 ng/ml), interleukin-6 (IL-6, 17.380 pg/ml), and brain natriuretic peptide (BNP, 260 pg/ml) were elevated and total albumin (28.1 g/L) was decreased. Renal function examination reported increased levels of α1-microglobulin (181.3 mg/L), β2-microglobulin (0.53 mg/L), microalbumin (1,071.3 mg/L), immunoglobulin G (68 mg/L), retinol binding protein (0.13 mg/L), N-acetyl-β-D-glucosamine (70.1 U/L), and transferrin (84 mg/L). The 24 h urine protein quantification was 289.00 mg. Fecal calprotectin (>1,800 ug/g) was notably promoted. Abdominal ultrasound and CT showed swollen and thickened ileocecal and colon walls and enlarged lymph nodes (Figure 2A). Thoracic high-resolution CT (HRCT) reported bronchiectasis and pneumonia mainly in the lingual lobe of the left lung and the middle lobe of the right lung (Figure 2A). Other examinations had no obvious abnormity.

**TABLE 1:** Blood examination.

Blood index	Results	Reference range
White blood cell (x109/L)	10.21	5.2–9.1
Platelet (x109/L)	280.00	100–300
Neutrophil (x109/L)	6.73	2–7
Lymphocyte (x109/L)	2.64	0.8–4
Eosinophil (x109/L)	0.24	0.05–0.5
Red blood cell (x1,012/L)	3.29	4–4.5
Hemoglobin (g/l)	91	120–170
Reticulocyte	0.029	0.005–0.015
CD3^+^ (%)	71.2	61.7–77
CD3^+^CD4^+^ (%)	36.4	25.8–41.6
CD3^+^CD8^+^ (%)	29.0	18.1–29.6
CD3^−^CD16^+^/CD56^+^ NK (%)	1.5	10.4–19.78
CD3^−^CD19^+^ B (%)	26.1	9.02–14.1

**FIGURE 3:**
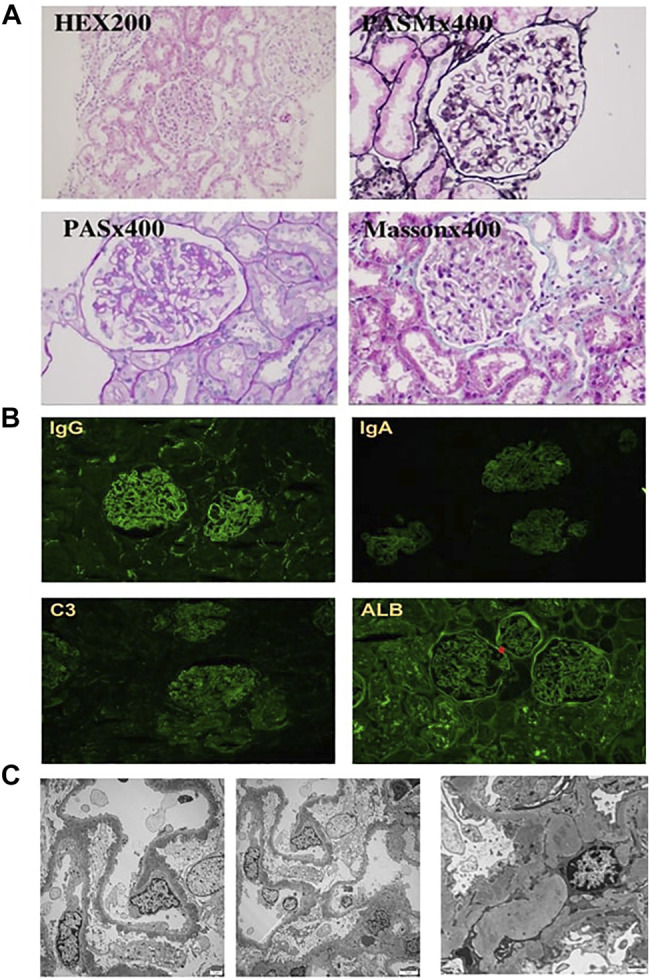
Light and electron microscopy findings and results of immunohistochemistry studies. **(A)** HE staining did not detect any crescent, PASM staining revealed spike-like structures deposited in the basement membrane, and Masson staining reported eosinophilic deposition in the basement. **(B)** Immunoglobulin (Ig) G, Ig A, C3, and albumin deposits identified at the glomerular capillary wall by immunohistochemical staining. **(C)** Electron microscopy confirms the diagnosis of MN. Magnification: **(A,B)** ×200–400, **(C)** ×2,500.

We made the primary diagnosis as proteinuria, inflammatory bowel disease (suspected), chronic otitis media, bronchiectasis, and autoimmune hemolysis. The application of antibiotics ceftazidime and ceftizoxime did not ameliorate the diarrhea, external auditory canal purulence, proteinuria, and other symptoms. In order to clarify the diagnosis, we performed renal biopsy under the guidance of ultrasound. Pathologic examinations including immunofluorescence (IF), electron microscope, and histological examinations were conducted, in which IF revealed IgG (+++), IgA (±), IgM (+), C3 (++), C1q (+), Fib (−), ALB (+), IgG1 (+++), IgG2 (±), IgG3 (±), IgG4 (++), PLA2R (±), and THSD7A (−) granule deposition (Figure 3B). Electron microscopy showed that the basement membrane of glomerular capillary loop was irregularly thickened, about 240–1,100 nm (Figure 3C); segmental proliferation of mesangial cells and stroma in the glomerular mesangial area; and podocyte foot process diffuse fusion (>80%). There were no obvious proliferation of parietal cells of renal capsule and no crescent formation. A large number of electron dense deposits in the basement membrane and under the epithelium of the glomerular capillary loop are accompanied by basement membrane reaction. A small amount of electron dense deposits can be seen around the transparent area, and electron dense deposits can be seen in the segmental glomerular mesangial area. Under a light microscope, the appearance of capillary loop was rigid, the segmental mesangial area was slightly widened, and there was no obvious mesangial cell proliferation; PASM staining revealed spike-like structure on the epithelial side of basement membrane of glomerular capillary loop; Masson staining reported eosinophilic deposition of subcutaneous eosinophils on the basement membrane of glomerular capillary loop (Figure 3A). We made the diagnosis of membranous nephropathy based on the following findings: the nail-like structure of glomerular basement membrane and the deposition of upper and subcutaneous erythrophils under the light microscope, the irregular thickening of basement membrane and the deposition of a large amount of electron dense matter under the upper and subcutaneous skin with basement membrane reaction under the electron microscope, and the fine granular deposition of IgG and C3 along the glomerular capillary loop by immunofluorescence. Considering that the concentration of PLA2R in blood is negative, we finally diagnose it as secondary membranous nephropathy.

Regarding his diarrhea, we performed colonoscopy and gastroscopy under anesthesia. Colonoscopy revealed particulate hyperplasia and submucosal hemorrhage in the terminal ileum, longitudinal ulcer in the sigmoid colon and descending colon, and erosive mucosa in the rectum (Figures 2B,C). Gastroscopy did not report any significant pathological changes. Biopsy indicated no obvious inflammatory changes existed in the terminal ileum, duodenum, or stomach, while chronic inflammatory cells in the stroma of large intestine mucosa increased, and the goblet cells decreased or disappeared. The focal crypt was distorted, a large amount of crypt inflammation and a small amount of crypt abscess were seen, and the apoptosis of crypt epithelium increased. Therefore, we made the diagnosis of inflammatory bowel disease based on these results.

However, expectant and supportive treatments failed to improve his symptoms. Since antibiotics had no significant efficacy, we intravenously injected 48 mg methylprednisolone, which slightly ameliorated his cough, while diarrhea, bloody purulent stool, and purulent auditory canal retained. In regard to autoimmune diseases such as systemic lupus erythematosus (SLE), we treated him with tacrolimus (1.0 mg at morning and 0.5 mg at evening) and 40 mg valsartan, which improved his cough, diarrhea, and proteinuria. To find the etiology, with the approval of parents, the peripheral blood of the boy and his parents was collected for whole-exon sequencing. Whole-exome sequencing yielded a mean of 10 Gb data with more than 99% coverage of the target region. Compared with the reference sequence (hg19), a missense variant, located in chr X:4 9 11 1 9 36, was detected in exon 8 of *FOXP3* (c.770A > C (p.Gln257Pro); N M _0 1 4 0 09) (Figures 1B,D) in both the boy and his mother. The variant was predicted to be pathogenic by all bioinformatics tools (Provean, SIFT, Polyphen2, mutationtaster, M-CAP, and REVEL); among them, Polyphen2 predicts that the pathogenicity score of the mutation at this site is 0.986, and mutationtaster predicts that the pathogenicity is disease causing. The variant has not been reported so far in ExAC, 1000 Genomes, and gnomAD. Structurally, the polypeptide surrounding the Gln257 residue is conserved across species. Sager sequencing showed the variant was not carried by his younger brother. We confirmed that the mutation segregated with IPEX within the pedigree, as illustrated in the pedigree. After discharge, the patient continued to receive the treatment of prednisone and tacrolimus and receive regular follow-up every 1 month. A recent follow-up suggested that his diarrhea, cough, alopecia areata, bilateral auditory canal pus, and hearing loss were significantly improved. Urinary protein fluctuated between “−” and “+,” and hemoglobin and liver and kidney function were in the normal range. During the follow-up period, there was a soft tissue infection of the right lower limb, which recovered after guiding intravenous drip of antibiotics. The mother and the boy were satisfied with the current treatment effect and had no intention of stem cell transplantation.

## Discussion

In recent years, although IPEX syndrome is characterized by inflammatory bowel disease, type I diabetes, and skin diseases, the number of IPEX cases exhibiting atypical symptoms was increasing (Bacchetta et al., 2018). The onset of IPEX in most children usually occurs within a few months after birth (Luo et al., 2018), even during the fetal period (Xavier-da-Silva et al., 2015). In our case, the patient suffered from autoimmune hemolysis, bronchiectasis, chronic otitis media, alopecia, relapse diarrhea, and proteinuria. These symptoms were not the primary symptoms of IPEX syndrome. Therefore, through this case, we recognize that the consideration of IPEX syndrome is important when a child develops multiple system disorders.

IPEX syndrome was firstly reported by Powell et al. as an X-linked recessive inherited disease since 19 males were found to have IPEX syndrome in a family. As a rare immunodeficiency syndrome, the bowel disease, endocrine disorder, and skin damage are the more common clinical manifestations. As physicians of nephrology, we paid more attention to the proteinuria of the patient. Therefore, we performed renal puncture biopsy that indicated membranous nephropathy. The expression of PLA2R in biopsy tissue was weakly positive, and that in serum was negative. Besides, the patient exhibited multiple system disorders, and there was no indication of autoimmune disease. Therefore, we performed whole-exon sequencing which confirmed the diagnosis of IPEX syndrome. In 2018, a multicenter study reported that 25% of 30 children from 25 families developed renal disease, with a median age of 3 years (1.8–9 years), in which 5 of them developed interstitial nephritis (three cases were probably linked to cyclosporine medication and one case developed chronic renal failure) (Duclaux-Loras et al., 2018a). Abir Bousset et al. (Boussetta et al., 2021) revealed that 19% of IPEX and IPEX-like syndromes would develop renal impairment, in which membranous nephropathy was the most common subtype. Other possible renal disorders included tubulointerstitial nephritis, focal tubular atrophy, minimal change disease, and irregular granular immune deposits in glomeruli and tubular basement membranes (Sheikine et al., 2015). Chuva et al. (2017) reported a case of PLA2R-positive membranous nephropathy in a child with IPEX syndrome, which indicated that PLA2R might be involved in renal impairment. However, they did not screen membranous nephropathy–related genes. In our case, the patient had isolated proteinuria, renal pathology suggested membranous nephropathy, and PLA2R was weakly positive in biopsy tissue, which might support the hypothesis of Teresa Chuv et al. that PLA2R might correlate with renal diseases. Meanwhile, the weakly positive expression of PLA2R in this case might be due to heterogeneity of the IPEX syndrome that different mutation sites caused different clinical symptoms.

A previous study reported more than 70 pathogenic variants in IPEX syndrome, in which 40% of variants located at the C-terminal forkhead (FKH) domain, 23% at the N-terminal proline-rich (PRR) domain, 14% at the leucine-zipper (LZ)-FKH loop, and 9% at the LZ domain (Barzaghi et al., 2018). It remained controversial to correlate the genetic variants with clinical manifestations. Duclaux-Loras et al. reported that clinical symptoms of IPEX syndrome had no significant association with the genetic variant in the FKH domain of FOXP3 gene (Duclaux-Loras et al., 2018b). They hypothesized that environmental exposures might modify the course of disease, leading to the varied manifestations in patients with the same mutation. In contrast, Jae HR et al. performed a systematic review that indicated the association between FOXP3 mutations and clinical manifestations: mutations in exon 1 and intron 1 of the N-terminal PRR domain were associated with infection-associated symptoms, which might be due to the mutation of a canonical LxxLL motif that could suppress the development of Th17 cells through the inhibition of retinoid-related orphan receptor-α (RORα)-mediated transcriptional activation (Du et al., 2008; Cohen et al., 2011); mutations in the LZ domain would reduce the suppressive activities mediated by Treg cells (Ziegler, 2006; Kinsey et al., 2009) and lead to Th2 cell–related diseases such as allergic rhinitis and asthma (Chatila et al., 2000; Bacchetta et al., 2006); mutations in the FKH domain would ameliorate the inhibition of transcriptional factors NFAT and AP1, which exaggerated immune responses (Wu et al., 2006); mutations in exon 11 in the FKH domain presented hematological, skin, and bowel diseases (Park et al., 2020); mutations in the ZF domain were primarily regarded to correlate with diarrhea and autoimmune hemocytopenia (Park et al., 2020). For patients with the same mutations but different manifestations, gene modification, epigenetic modification, and environmental factors were considered the contributors. In our case, we found a novel mutation in exon 8 c.770A > C (p.Gln257Pro) in the LZ domain. However, its manifestations were not consistent with the previous description (immune disorder). The primary onset of our case was autoimmune hemolysis and bronchiectasis. Diarrhea and proteinuria arose in recent years. Therefore, the correlation between mutation sites and clinical manifestations required additional investigation. In addition, the disease course in our case lasted more than 10 years, which raised the difficulty to diagnose IPEX syndrome. Our case would provide a reference for the diagnosis of IPEX syndrome that the previous history of different system impairment should be combined and taken into consideration.

Current treatment for IPEX syndrome is supportive strategies regarding clinical symptoms (Torgerson and Ochs, 2007). Early diagnosis and intervention remain to be the most important strategy to improve the prognosis. Hematopoietic stem cell transplantation presents to be the only effective approach to cure IPEX syndrome (Mazzolari et al., 2005; Rao et al., 2007). Moreover, the immunosuppressive drugs such as cyclosporine A or FK506 have been successfully used but only effective in some patients (Ferguson et al., 2000; Levy-Lahad and Wildin, 2001; Wildin et al., 2002). In our case, the patient received the treatment of glucocorticoid and tacrolimus, which successfully ameliorated the diarrhea, infection, and kidney dysfunction. Therefore, our study proposed the tailored and appropriate application of immunosuppressive drugs for the treatment of IPEX syndrome.

## Data Availability

The original contributions presented in the study are included in the article/supplementary material, and further inquiries can be directed to the corresponding authors.
